# Expression of Cell-Cycle Regulatory Proteins pRb, Cyclin D1, and p53 Is Not Associated with Recurrence Rates of Equine Sarcoids

**DOI:** 10.3390/vetsci9090474

**Published:** 2022-09-01

**Authors:** Giorgia Tura, Barbara Brunetti, Elena Brigandì, Riccardo Rinnovati, Giuseppe Sarli, Giancarlo Avallone, Luisa Vera Muscatello, Roberto Marcello La Ragione, Andy E. Durham, Barbara Bacci

**Affiliations:** 1 Department of Veterinary Medical Sciences, University of Bologna, 40064 Bologna, Italy; 2 School of Veterinary Medicine and Science, University of Surrey, Guildford GU2 7XH, UK; 3 School of Biosciences and Medicine, University of Surrey, Guildford GU2 7XH, UK; 4 Liphook Equine Hospital, Liphook GU30 7JG, UK

**Keywords:** equid, sarcoid, bovine papillomavirus, p16^CDKN2A^, pRb, Cyclin D1, p53, Ki67

## Abstract

**Simple Summary:**

Expression of cell-cycle regulatory proteins pRB, Cyclin D1, and p53 is not associated with recurrence rates of equine sarcoids. Sarcoids are common tumors of equids, and since the pathogenesis and Bovine Papillomavirus (BPV) mechanism of tumorigenesis is not fully understood yet, we investigated the expression of cell cycle regulatory proteins pRB, Cyclin D1, and p53 to understand whether BPV and Human Papillomavirus (HPV) share similar mechanisms of action. The expression pattern of these proteins in a series of BPV-positive equine sarcoids is different from HPV-induced tumors. Moreover, their expression is not correlated with the recurrence or occurrence of sarcoids in new body sites.

**Abstract:**

Sarcoids are among the most common tumors diagnosed in equids; their association with bovine papillomaviruses (BPV) infection has been widely reported, but the mechanism of carcinogenesis has not been fully elucidated. To verify whether BPV infection causes dysregulation of the pRb-Cyclin D1-p16^CDKN2A^-p53 pathway as reported for human papillomavirus (HPV), the study employed immunohistochemistry to test 55 equine sarcoid biopsies for the expression of pRb, Cyclin D1, and p53 cell cycle regulatory proteins and to evaluate the proliferative rate through Ki67. High Cyclin D1 and pRb expression were observed in 51% and 80% of cases, respectively, while low expression was observed in 49% and 20% of cases, respectively. Significantly higher Ki67 proliferation indexes were observed in fibroblastic, nodular, and mixed sarcoids compared to the occult and verrucous. High proliferation was significantly associated with high Cyclin D1 expression. In contrast with previous studies, p53 positivity was not observed in the cases examined in this study. Moreover, follow-up analysis revealed that fibroblastic, mixed sarcoids were associated with significantly higher local recurrence rates while the verrucous subtype was associated with higher rates of new sarcoid development at distant sites.

## 1. Introduction

Sarcoids are the most common cutaneous tumors that affect equids worldwide [1,2,3]. These tumors are locally invasive, non-metastatic, and mostly non-regressing [4,5]. There is considerable evidence that the development of sarcoids is associated with bovine papillomavirus (BPV) infection [6,7]. In equine sarcoids, BPV E5 binds and activates PDGFR-β in a ligand-independent manner, inducing receptor oligomerization, autophosphorylation, and activation. In contrast, oncogenic human papillomaviruses (HPV) produce the oncogenes E6 and E7, which inactivate the tumor suppression genes p53 and pRb. As a result, p16^CDKN2A^ is overproduced as a by-product of the inactivation pRb [8]. In human papillomavirus (HPV) infection the virus interferes with the p16^CDKN2A^ -pathway, but this mechanism of action has not been demonstrated in equine sarcoids, where the main mechanism of action is linked to dysregulation of the PDGFRβ receptor [9,10].

The expression of these cell cycle proteins in equine sarcoids is not yet known and in general, the analysis of cell cycle regulatory proteins in equine sarcoids is very limited. p53 expression has been previously investigated and found to be abnormally expressed in various studies [11,12,13]. In addition, the expression of cell cycle regulatory proteins Cyclin A, CDK2, and p27^kip1^ was investigated previously, but BPV did not seem to have a major influence on the expression of these molecules [14]. Previous studies have demonstrated that the BPV E7 protein lacks the canonical pRB binding motifs associated with the development of fibropapillomas [15,16]. In contrast, the involvement of cell cycle proteins of the pRb-Cyclin D1 has not been investigated in BPV-induced sarcoids.

Hence, to establish a possible interaction between BPV and cell cycle regulatory proteins, the study reported here aimed to assess the expression of cell cycle proteins p53, pRb, Cyclin D1 as well as proliferation index through Ki67 analysis in a series of confirmed BPV-positive equine sarcoids. The results will also be correlated with morphological histotypes and follow-up data.

## 2. Materials and Methods

### 2.1. Case Selection and Study Design

An observational cohort study was conducted on fifty-five (55) sarcoids selected from the pathology archives of the University of Bologna (Italy) and the University of Surrey (UK) between 2017 and 2020. The diagnosis was confirmed by histology, in situ hybridization (RNAscope) for BPV-1/2, and PCR for BPV-1/2/13 as part of a previous investigation [17]. In brief, 53/55 cases in the study were positive for BPV-1 at PCR, and 53/55 had cytoplasmic and nuclear hybridization signals in neoplastic fibroblasts. All cases were positive at either RNAscope or PCR. Clinical data, including signalment and sarcoid clinical type, were also available (Table S1). Formalin-fixed paraffin-embedded (FFPE) and Tissue microarrays (TMA) samples were available for all cases [17].

### 2.2. Immunohistochemistry

Immunohistochemistry for p53, pRb, Cyclin D1, and Ki67 was performed on either full sections (p53, pRb, Ki67) or tissue microarrays (TMA) (Cyclin D1) which were prepared as previously described [17]. Three-micrometer-thick sections were dewaxed and rehydrated. For antigen retrieval, sections were immersed in 200 mL citrate buffer (pH 6.00) or in tris-EDTA (pH 9.00) and p16 immunostaining, respectively. Enzymatic antigen retrieval was performed in 37 °C oven for 15 min with 0.05% trypsin. Slides were incubated overnight at 4 °C with the following primary antibodies: Ki67 (mouse monoclonal, clone MIB-1, Dako Denmark, dilution 1:600); p53 (mouse monoclonal, clone PAb240, BD Pharmigen, 1:100 dilution), pRb (mouse monoclonal, clone IF8, Santa Cruz Biotechnology, dilution 1:100), and Cyclin D1 (mouse monoclonal, clone A12, Santa Cruz Biotechnology, dilution 1:200). The reaction was developed using a commercial streptavidin-biotin-peroxidase technique (ABC kit, Vector, Burlingame, CA, USA) and visualized with 3-amino-9-ethylcarbazole (Dako, Glostrup, Denmark). Slides were counterstained with Meyer hematoxylin. Equine tissue samples previously shown to be p53 overexpressors (squamous cell carcinoma) were used as a positive control. For pRb and Cyclin D1 normal testis and skin were used as control. For Ki67, equine lymph node was used as positive control. As negative controls, the primary antibody was replaced with an isotype-matched non-relevant antibody.

Cyclin D1 and pRb were evaluated for the percentage of positive cells (0: no positivity, 1: <25%, 2: 25–50% and 3: >50%). Scores 0 and 1 of pRB/Cyclin D1 were considered as low to normal), while scores 2 and 3 were considered as overexpression [18]. For p53 cases were considered positive when at least 10% of neoplastic cells had nuclear positivity. For Ki67 the number of positive neoplastic cells was counted in five 400x fields (including at least 500 cells). Cells were counted within the tumor areas characterized by the highest number of positive cells. Two veterinary pathologists (BB, GT) independently scored all cases and any discrepancies were resolved by re-scoring on a multi-headed microscope.

### 2.3. Western Blotting

Cross reaction of antibodies for pRb and CyclinD1 with equine tissues was not yet validated. To this aim, western blotting was performed to confirm the specificity of antibodies for pRb and Cyclin D1 with equine tissues.

Tissue lysates of fresh equine testis and sarcoid were used. From each, 25 µg of tissue was homogenized with 500 mL RIPA buffer (Thermofisher Scientific, Waltham, MA, USA. Tubes were centrifuged at 12,000 rpm or 30 min at 4 °C and the supernatant collected and stored at −80 °C. Protein concentration was calculated using the Bradford protein Assay Kit (Thermofisher Scientific). Subsequently, 40 µg of proteins were denatured at 100 °C for 5 min. Proteins were separated using a 10% gel and electrophoretically transferred to a nitrocellulose membrane. Non-specific binding sites were blocked with BSA 5% in TBS-T for 1 h at RT. The blot was cut and part was incubated at 4 °C overnight part with an antibody against pRB (1:500, Santacruz Biotechnology) and part with Cyclin D1 (1:500, Santacruz Biotechnology). Then, the membrane was incubated with anti-mouse HRT-conjugated secondary antibody (1:200, Thermofisher Scientific) for Cyclin D1 and pRB and with anti-rabbit HRT-conjugated secondary antibody (1:200, Thermofisher Scientific) for 1 h at room temperature. Reactive bands were visualized with a chemiluminescent detection kit (Pico Thermofisher Scientific) using the Chemidoc instrument (Bio-Rad, Hercules, CA, USA). Βeta-Actin was used as the loading control and was detected using a β-actin-specific antibody (1:500, Santa Cruz Biotechnology, Dallas, TX, USA).

### 2.4. Follow-Up

Follow-up information was retrieved by telephone interview with the animal owners or referring veterinarians. Recurrence was defined as either recurrence at the same site (local recurrence, LR) of previous sarcoid and occurrence of a distant sarcoid (de novo occurrence, DNO). These were recorded as present or absent. Time to recurrence was calculated as days elapsed from the histological diagnosis to LR and DNO. Cases in which no LR or DNO was recorded at the time of the study were censored.

### 2.5. Statistical Analysis

Normal distribution of continuous variables was assessed using the Kolmogorov-Smirnov test of normality. Mean ± standard deviation was indicated for normal continuous variables, whereas the median and range were calculated for non-normally distributed continuous variables. Distribution of categorical variables was assessed with the Chi-square test. To assess the distribution of continuous variables, Kruskal–Wallis and one-way ANOVA tests (with Bonferroni pairwise comparison) were used. For follow-up analysis of tumor recurrence, Kaplan–Meier survival curves and log-rank test were performed. For each test, differences were considered significant when *p* ≤ 0.05. All statistical analysis was performed using R (version 4.1.2 for Windows, Vienna, Austria).

## 3. Results

### 3.1. Population Characteristics

A total of fifty-five (55) cases of sarcoids from an equal number of equids were analysed. The subjects were 51 horses and 4 donkeys. Most represented equine breeds were Warmbloods (*n =* 6), Irish Sports (*n =* 5) and Thoroughbred (*n =* 5). Subjects were 30 males (22 geldings) and 20 females; in 5 cases sex was unknown. The mean ± standard deviation age was 8 ± 3.8 years. Sarcoids were located in the head and neck (*n =* 18), genital (*n =* 14), trunk (*n =* 15) and limbs (*n =* 7) and one case was unknown. Clinical types were nodular (*n =* 29), fibroblastic (*n =* 9), verrucous (*n =* 9), mixed (*n =* 5) and occult (*n =* 3). In ten horses sarcoids were multiple (number of sarcoids ranging from 1 to 6 each). Only one sarcoid was tested for each equid.

### 3.2. Western Blotting and Immunohistochemistry

**Figure 1:**
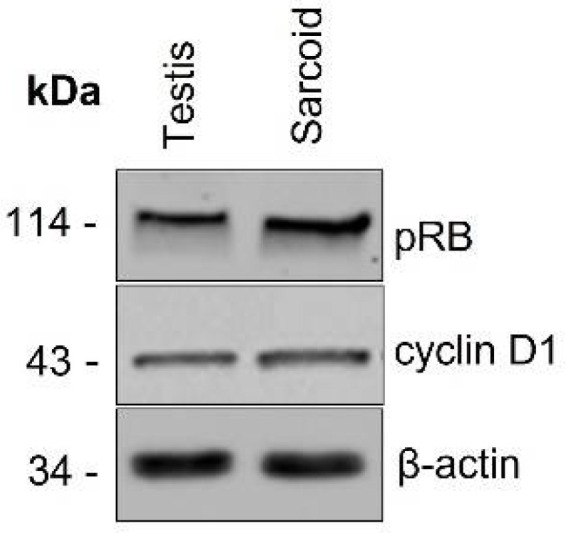
Western blot. Western Blot analysis shows 43 KDa and 114 KDa bands in both normal equine testis and sarcoid, corresponding to Cyclin D1 and pRb protein, respectively. Beta-actin was used as control.

Western bot analysis confirmed cross-reactivity of the pRB and Cyclin D1 antibodies for equine tissues (Figure 1).

Immunohistochemical results are summarized in Table 1.

Examination of control tissues was performed for each antibody: in the testis spermatogones were positive for pRb; Leydig cells and Sertoli cells were positive for Cyclin D1 (Figure S1). Rb-positive cells were observed in the basal and suprabasal layers of the epidermis and Cyclin D1-positive cells were limited to the basal layer and bulbar cells in the hair follicles (Figure S2). Positive control for p53 consisted of an equine squamous cell carcinoma known to overexpress the protein.

**Figure 2:**
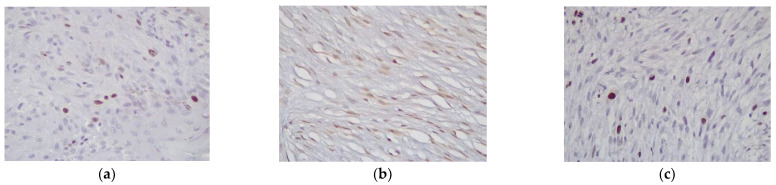
Horse, sarcoid. Immunohistochemistry for (**a**) pRb antibody showing strong nuclear positivity in neoplastic fibroblasts (score 1) (Ob.200×) (**b**) Cyclin D1 showing moderate nuclear positivity in the majority of neoplastic cells (score 3) (Ob.100×) (**c**) Ki-67 showing a minority of cells with nuclear positivity (Ob.200×).

Immunohistochemistry for pRb was performed on all cases. Of these, 28 cases had a score of 1, 17 had a score of 2, and 10 cases had a score of 3. Positivity was exclusively nuclear (Figure 2a). pRb scores were low in 28 cases (51%) and high in 27 cases (49%). Cyclin D1 was positive in all cases; 11 cases had a score of 1, 22 cases had a score of 2, and 22 cases had a score of 3, hence 11 cases were classified as low (20%) and 44 cases as high (80%)(Figure 2b). Positivity was exclusively nuclear. All cases were negative for p53. Ki67 expression was evaluated in all cases with a median Ki67 value of 5.4% (range 1–20%) (Figure 2c).

Cyclin D1 and pRb scores were positively correlated (Chi-square test, *p* = 0.03). Ki67 index positively correlated with Cyclin D1 (one-way ANOVA, *p* = 0.008), but not with pRb expression (one-way ANOVA, *p* = 0.44).

Immunohistochemical results were also compared with selected clinical variables.

Of the cell cycle proteins investigated, pRb scores were similar across these groups. By contrast, Cyclin D1 scores were significantly higher in the fibroblastic, nodular, and mixed subtypes (Chi-square test, *p* = 0.006). Ki67 proliferation index was also significantly different in the five groups, with the fibroblastic and nodular sarcoids having significantly higher proliferation rates than the occult and verrucous type (One-way ANOVA, *p* = 0.03, Supplementary Table S1). In addition, cases were dichotomized into high and low proliferation using the median Ki67 value (5.45). With this method, 27 cases were classified as “low” and 28 cases as “high”. High proliferation cases were significantly correlated with the fibroblastic, nodular, and mixed subtypes (Chi-square test, *p* = 0.021).

### 3.3. Follow-Up

**Figure 3:**
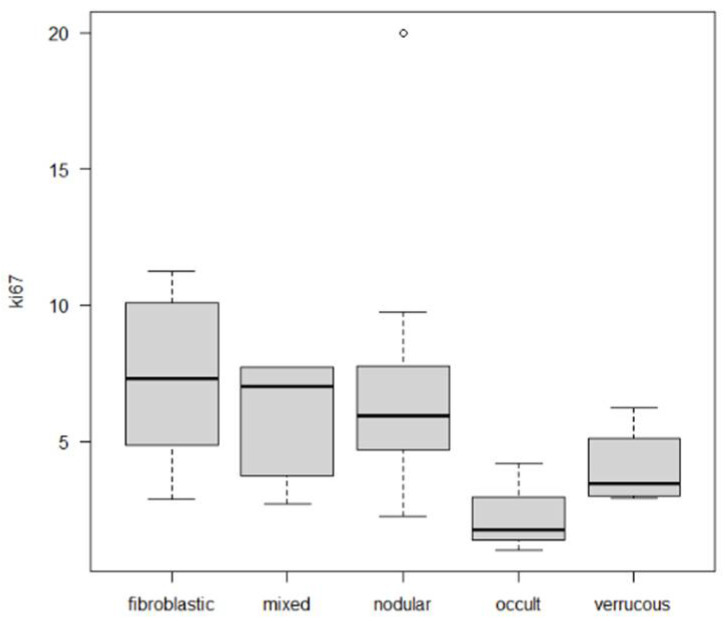
Distribution of Ki67 index in sarcoid clinical subtypes. The fibroblastic, mixed, and nodular subtypes have significantly higher proliferation indexes compared to the occult and verrucous types. (One-way ANOVA, *p* = 0.021).

Follow-up information was available for 30 cases. In 14 cases horses developed new sarcoids, either locally (LR, *n =* 9, 30%) or at a different body location (de novo occurrence *=* DNO, *n =* 5, 16.6%). In 16 cases horses did not develop new sarcoids. Median time to LR days (95% CI 188-na). LR and DNO were significantly associated with the clinical type of sarcoids (Chi-square test *p* < 0.01). In general, fibroblastic and mixed sarcoids were associated with LR (log-rank test, *p* < 0.0001 while the verrucous type was significantly associated with de novo occurrence (DNO) of other sarcoids in other body sites (log-rank test, *p =* 0.009) (Table 2). In detail, of the cases with known follow-up, 7/8 fibroblastic and 2/2 mixed sarcoids recurred at the same site; 4/5 verrucous subtypes did not recur locally but new sarcoids developed in distant sites while only one of 12 nodular sarcoids in the study developed a new sarcoid at a distant site. Overall, these differences were statistically significant (Chi-square test, *p* < 0.01). None of the cell cycle proteins investigated correlated with either LR or DNO (Table 3), although cases with high Cyclin D1 scores had a higher number of recurrences, which, however, were not statistically significant (Figure 3).

## 4. Discussion

The components of the p16^CDKN2A^ -Cyclin D/CDK-pRb pathway (G1 pathway) are frequently altered in various types of human cancers. When activated by Cyclin D1, CDK4 is able to phosphorylate pRb, leading to the release of associated proteins including E2F which has the ability to activate the genes necessary for cell progression through the G1 phase. p16^CDKN2A^ controls proliferation during G1 by inhibiting the ability of Cyclin D/CDK4 and Cyclin D/CDK6 complexes to phosphorylate pRb [18,19]

Since it is not known whether BPV interacts with cell cycle molecules, the study of cell cycle regulators Cyclin D1, pRb, and p53 in equine sarcoids may elucidate the mechanisms of BPV-mediated tumorigenesis.

The expression of p16^CDKN2A^ could not be assessed in this study, due to the lack of cross-reactivity of the commercially available human antibodies with equine tissue (results not shown).

In HPV-induced tumors, pRb is degraded by HPV resulting in p16^CDKN2A^ overexpression and pRb loss [20,21]. pRb loss is associated with poor prognosis in several types of human tumors [22,23]. In HPV-associated tumors, there is an inverse correlation between HPV presence and pRb expression and in tumors exhibiting abnormal p16^CDKN2A^ cytoplasmic expression, concurrent pRb loss is observed [24]. The expression of pRb in all sarcoids demonstrates that despite its role as a tumor suppressor, neoplastic cells retain pRb expression similarly to normal tissues. In fact, in 49% of cases, a high percentage of neoplastic cells was strongly pRb positive. This potentially indicates that BPV does not cause pRb loss but may also suggest that pRb may support tumorigenesis through other mechanisms [25]. However, it must be highlighted that the antibody used in this study to detect pRb does not distinguish between the phosphorylated and unphosphorylated forms. Therefore, the protein activation status is unknown.

Cell proliferation was investigated with the Ki67 index to verify if higher proliferation rates were associated with different behavior. Although a significantly higher expression was found in fibroblastic and nodular sarcoids, this parameter did not show any correlation with behavior, in fact, survival analysis revealed that the Ki67 index was not correlated with recurrence.

Cyclin D1 expression has also been investigated in various human tumors and found to be overexpressed in several tumor types [26]. In general, overexpression of Cyclin D1 is associated with higher-grade neoplasms and poorer outcomes [27]. Overexpression of cyclin D1 protein is found in many human tumors such as mantle cell lymphoma, non-small cell lung cancer, plasma cell myeloma, hairy cell leukemia, as well as breast and esophageal cancers [28]. In veterinary species, its expression was investigated in canine melanoma and found to correlate with proliferation index [29]. In papillomavirus-induced tumors, Cyclin D1’s role is less clear and in particular, in animal species with papillomavirus-associated neoplasms, its role in tumorigenesis is not known. In this study, Cyclin D1 was found to be overexpressed in the majority of cases (80%) and although it was not associated with recurrence, it was associated with higher tumor proliferation rates. Interestingly, the highest expression was seen in the highly proliferative subtypes, the nodular and fibroblastic and hence it was higher in sarcoids with higher recurrence rates; although not prognostically relevant, Cyclin D1 may represent an additional marker of proliferation.

Previous studies have also reported abnormal p53 expression in sarcoids [11,14]. It is known that the E6 oncoprotein of both HPV and BPV affects p53, but the mechanisms that induce the reduction levels of this tumor suppressor protein are distinct between these PVs types. The E6 oncoprotein of HPV binds to the E6AP ubiquitin ligase, resulting in p53 ubiquitination and proteasomal degradation. In contrast, the BPV E6 oncoprotein does not induce p53 degradation but interacts with CBP/p300, promoting the downregulation of p53 [30]. In the study reported here, none of the cases demonstrated p53 positivity, in contrast with previous investigations, in which p53 positivity was observed in 10–44% of cases [13,14]. These results indicate that p53 mutations (and subsequent accumulation as detected by immunohistochemistry) in sarcoids are less likely but may also indicate that degradation occurs similarly to what is observed in HPV infection.

Follow-ups of cases were available in a subset of the cases included in this study. A proportion of cases recurred locally (30%) while in a small proportion of cases new sarcoids developed at distant sites (16.6%). The cell proteins investigated did not predict behavior while recurrence was significantly different according to the clinical type. The fibroblastic and mixed subtypes tended to recur locally, the verrucous subtype was associated with the occurrence of new sarcoids at other body sites, and the nodular and occult sarcoids were not associated with recurrence. Recurrence data are available in the literature and are reported to be around 15–20% depending on treatment types [31]; in this study, recurrence rates were higher, but this may be influenced by case selection bias. Previous studies on sarcoid treatment have reported slightly lower recurrence rates for verrucous sarcoids [31].

In conclusion, clinical types of sarcoids appear to have different clinical outcomes, with fibroblastic, mixed, and verrucous sarcoids demonstrating higher recurrence/de novo occurrence rates. Moreover, cell cycle proteins pRb and p53 do not appear to be dysregulated in BPV-induced equine sarcoid. Although Cyclin D1 is overexpressed in sarcoids with high proliferation rates and has higher scores in sarcoids that recurred locally or occurred de novo, this marker is not significantly associated with clinical outcomes.

## Figures and Tables

**Table 1:** Summary of immunohistochemical results based on clinical types of 55 sarcoids.

Protein	Score	Percent (%)	Fibroblastic *n*(%)	Mixed *n* (%)	Nodular *n* (%)	Occult *n* (%)	Verrucous *n* (%)	*p* Value
**pRb**	low (score 1)	51%	5 (55.6)	1 (20.0)	16 (55.2)	1 (33.3)	5 (55.6)	0.618
	high (score 2–3)	49%	4 (44.4)	4 (80.0)	13 (44.8)	2 (66.7)	4 (44.4)	
**Cyclin D1**	low (score 1)	20%	2 (22.2)	1 (20.0)	5 (17.2)	3 (100.0)	0 (0.0)	0.006 *
	high (score 2–3)	80%	7 (77.8)	4 (80.0)	24 (82.8)	0 (0.0)	9 (100.0)	
**Ki67** Median [95% CI]	5.45		7.30 [2.88, 11.25]	7.01 [2.71, 7.72]	5.95 [2.24, 20.00]	1.75 [1.00, 4.20]	3.45 [2.90, 6.24]	0.015 *
**Ki67**	low	49%	4 (44.4)	2 (40.0)	10 (34.5)	3 (100.0)	8 (88.9)	0.021 *
	high	51%	5 (55.6)	3 (60.0)	19 (65.5)	0 (0.0)	1 (11.1)	

* statistically significant.

**Table 2:** Follow-up information for 30 horses with sarcoids, according to the clinical type.

Clinical Type	LR	Median Tume to LR (95% CI)	DNO (Distant)	Median Time to DNO (95% CI)	No Recurrence
Fibroblastic (8)	7	145 (60–180)	0	-	1
Mixed (2)	2	150 (108-na)	^1^ 0	-	0
Nodular (13)	0	-	1	-	12
Occult (2)	0	-	0	-	2
Verrucous (5)	0	-	4	205 (95-na) ^1^	1

^1^ insufficient number of cases to calculate 95%CI.

**Table 3:** Follow-up information for 30 horses with sarcoids, according to cell cycle protein expression.

Cell Cycle Protein	Low	High	*p* Value
pRb	14	16	0.189
Cyclin D1	8	22	0.212
Ki67	12	18	0.664

## Data Availability

Data supporting reported results are available upon request.

## Supplementary Materials

The following supporting information can be downloaded at: https://www.mdpi.com/article/10.3390/vetsci9090474/s1, Figure S1: Immunohistochemical positivity of pRb and Cyclin D1 in the control (testis); Figure S2: Immunohistochemical positivity of pRb and Cyclin D1 in the control (skin); Table S1: One-way ANOVA and Bonferroni pairwise comparison between the Ki67 index of the different clinical types of sarcoids.
