# Supplementary material for: Expression of Cell-Cycle Regulatory Proteins pRb, Cyclin D1, and p53 Is Not Associated with Recurrence Rates of Equine Sarcoids
Source: Vet Sci. 2022 Sep 1;9(9):474. doi: 10.3390/vetsci9090474 (PMC9504470; doi:10.3390/vetsci9090474)
Supplement: Supplementary file 1 [file vetsci-09-00474-s001.zip › vetsci-1761402-supplementary.pdf]

Supplementary material

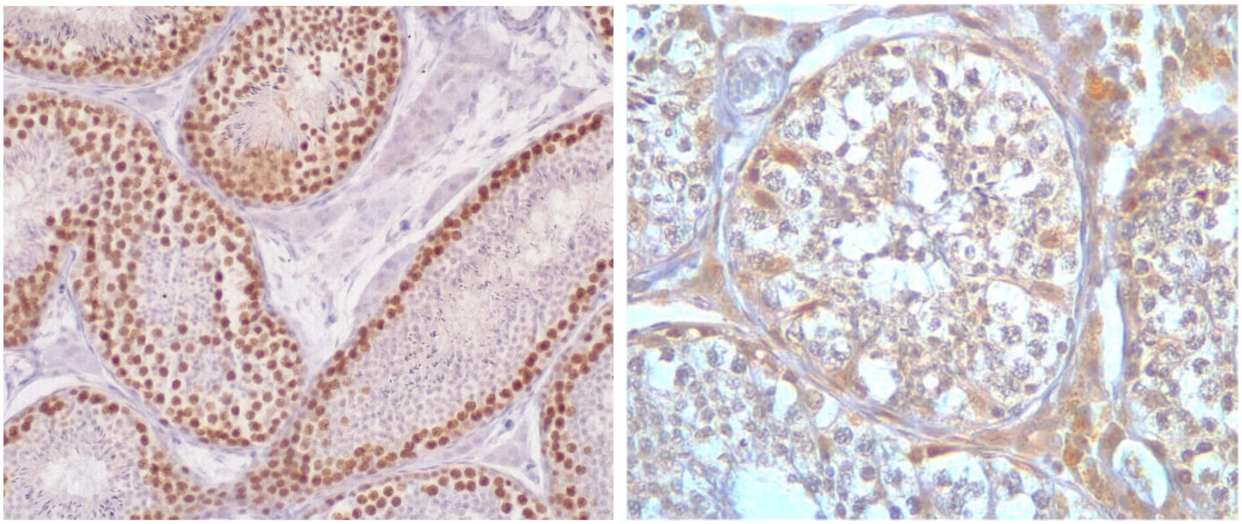

(**a**) (**b**)

Figure S1. Horse, testis. Immunohistochemistry showing **(a)** positivity for pRB in the nuclei of spermatogons in the seminiferous tubules (Ob. 400×) (**b**) strong nuclear positivity of Leydig (interstitial cells) and Sertoli cells in the seminiferous tubules (Ob.200×).

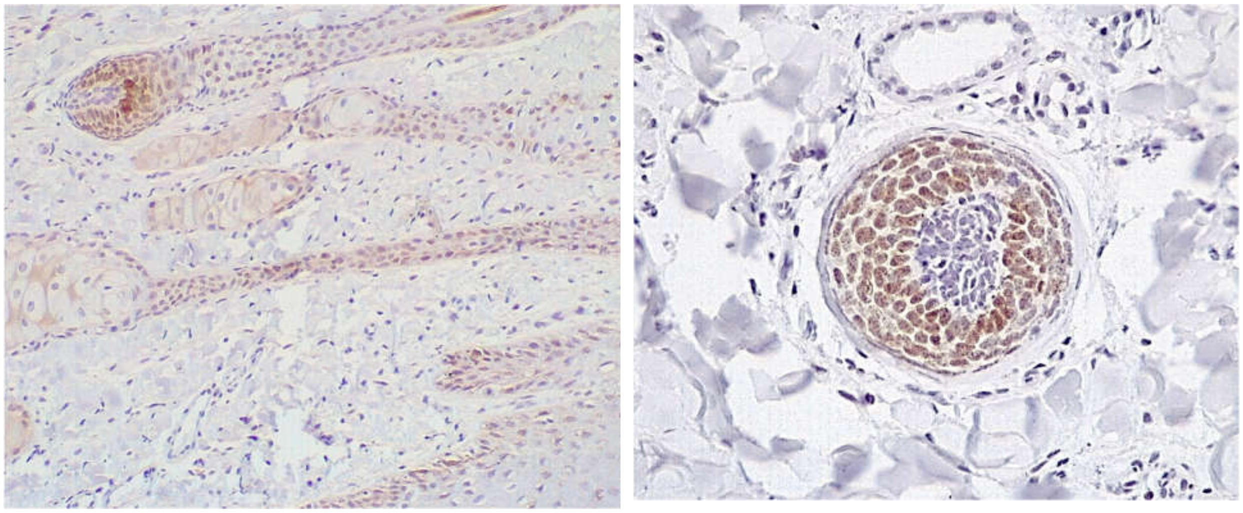

(**a**) (**b**)

Figure S2. Horse, skin. Immunohistochemistry for (**a**) pRB antibody showing strong nuclear positivity of basal cells of the epidermis and hair follicle (Ob.100×) (**b**) Cyclin D1 showing nuclear positivity in hair follicle cells (Ob.400×).

	fibroblastic	mixed	nodular	occult
mixed	1.00	-		
nodular	1.00	1.00	-	
occult	0.14	1.00	0.21	-
verrucous	0.28	1.00	0.37	1.00

Table S1. One-way ANOVA and Bonferroni pairwise comparison between the Ki67 index of the different clinical types of sarcoids (p=0.03) The fibroblastic and nodular sarcoids have significantly higher proliferation index than bothe the occult and verrucous sarcoids.
